# Temporal Changes in the Quality of Acute Stroke Care in Five National Audits across Europe

**DOI:** 10.1155/2015/432497

**Published:** 2015-12-09

**Authors:** Steffi Hillmann, Silke Wiedmann, Alec Fraser, Juan Baeza, Anthony Rudd, Bo Norrving, Kjell Asplund, Maciej Niewada, Martin Dennis, Peter Hermanek, Charles D. A. Wolfe, Peter U. Heuschmann

**Affiliations:** ^1^Institute of Clinical Epidemiology and Biometry, University of Würzburg, 97080 Würzburg, Germany; ^2^Comprehensive Heart Failure Center, University Hospital of Würzburg, 97080 Würzburg, Germany; ^3^Faculty of Public Health and Policy, London School of Hygiene and Tropical Medicine, London WC1E 7HT, UK; ^4^Department of Management, School of Social Science and Public Policy, King's College London, London SE1 3QD, UK; ^5^Division of Health and Social Care Research, King's College London, London SE1 1UL, UK; ^6^Department of Clinical Sciences, Neurology, Lund University, 221 00 Lund, Sweden; ^7^Department of Public Health and Clinical Medicine, Umeå University, Umeå 901 85, Sweden; ^8^Department of Experimental and Clinical Pharmacology, Medical University Warsaw, 02-091 Warsaw, Poland; ^9^Department of Clinical Neurosciences, Western General Hospital, Edinburgh EH4 2XU, UK; ^10^Bavarian Permanent Working Party for Quality Assurance, 80331 Munich, Germany; ^11^National Institute for Health Research Biomedical Research, Centre Guy's & St Thomas' NHS Foundation Trust and King's College London, London SE1 9RT, UK; ^12^Clinical Trial Center Würzburg, University Hospital Würzburg, 97080 Würzburg, Germany

## Abstract

*Background. *Data on potential variations in delivery of appropriate stroke care over time are scarce. We investigated temporal changes in the quality of acute hospital stroke care across five national audits in Europe over a period of six years.* Methods.* Data were derived from national stroke audits in Germany, Poland, Scotland, Sweden, and England/Wales/Northern Ireland participating within the European Implementation Score (EIS) collaboration. Temporal changes in predefined quality indicators with comparable information between the audits were investigated. Multivariable logistic regression analyses were performed to estimate adherence to quality indicators over time.* Results.* Between 2004 and 2009, individual data from 542,112 patients treated in 538 centers participating continuously over the study period were included. In most audits, the proportions of patients who were treated on a SU, were screened for dysphagia, and received thrombolytic treatment increased over time and ranged from 2-fold to almost 4-fold increase in patients receiving thrombolytic therapy in 2009 compared to 2004.* Conclusions.* A general trend towards a better quality of stroke care defined by standardized quality indicators was observed over time. The association between introducing a specific measure and higher adherence over time might indicate that monitoring of stroke care performance contributes to improving quality of care.

## 1. Introduction

In several countries, mainly from Europe and Northern America, stroke audits were implemented to provide information on the quality of acute hospital care for the local population [1–9]. In most of these audits, regular benchmarking activities were implemented by comparing measures of quality of care between the participating centers on a national or regional level [10].

Assessing potential trends in quality of stroke care over time within existing audits might be useful for identifying factors influencing changes in delivery of appropriate care on the population level. For example, specific strategies suspected to drive improvement in clinical practice and service development [11] could be linked with variations in quality of care observed within a given region. However, most previously published audit data did not provide distinct information of trends in quality of care over time [12–16]. There are only a few publications addressing temporal trends in quality of care [8, 9]. In addition, variations in methodology, data documentation, and variable definition of exiting audits in Europe currently hamper direct comparability of data from different national or regional audits [10]. Therefore, little data on time trends regarding delivery of appropriate stroke care in different countries or regions is available.

We investigated variations in quality of acute stroke care across five national audits in Europe using comparable variable definitions and assessed time trends in delivery of appropriate care over a period up to 6 years.

## 2. Methods

### 2.1. Data Collection

Data were derived from stroke audits participating within the European Implementation Score (EIS) project. The EIS Project is a European Union funded project (number 223153) aiming at developing a European methodology to assess the implementation of research evidence into practice. The selection and the characteristics of national or regional stroke audits participating in the EIS project have been described previously [8]. For the present analyses, audits providing datasets on more than two years of documentation during the study period 2004–2009 were included. Therefore, the Catalan Stroke Audit was excluded from the present analysis and the following stroke audits were included: the German Stroke Register Study Group [ADSR], Germany, the Hospital Stroke Registry of National Program for Prevention and Treatment of Cardiovascular Diseases [POLKARD], Poland, the Scottish Stroke Care Audit [SSCA], Scotland, the National Stroke Register in Sweden [Riks-Stroke], Sweden, and the National Sentinel Audit of Stroke [NSSA], England/Wales/Northern Ireland. An overview on the characteristic of the participating audits was published previously [17]. The study was limited to end at 2009 due to the end of the EIS project.

### 2.2. Data Definition

The following variables were documented in a comparable way over time across at least four of the five audits: demographics: age; sex; dependency prestroke (dependent, independent); stroke subtype (ischemic stroke [IS], intracerebral haemorrhage [ICH], and undefined [UND]); admission: time interval between onset and admission (≤3 h, ≥3 h/missing); day of admission (weekend; weekday); comorbidities/risk factors: atrial fibrillation [AF] (No/Yes); process of care: brain imaging (No/Yes); intravenous treatment with tissue-plasminogen-activator [rt-PA] (No/Yes); Stroke Unit [SU] treatment (No/Yes); testing for swallowing disorders (NO/YES or unassessable if documented); antiplatelet therapy during hospital stay (No/Yes); anticoagulant therapy during hospital stay or recommended at discharge (No/Yes); length of hospital stay (days); level of consciousness an admission or worst level during first week (awake/disturbed). The definitions of the variables involved in the present analysis were stable over the whole time period in the participating audits. A standardized codebook was developed in cooperation with audit representatives before the data were pooled. The recoding of the variables was subsequently verified by audit representatives. Results were also checked by comparing outputs of this analysis with publications (e.g., papers or reports) of the participating audits.

### 2.3. Quality Indicators [QI]

Based on these variables the following quality indicators were calculated with inclusion and exclusion criteria proposed by a European consensus group:* screening for dysphagia* (numerator: number of stroke patients screened for swallowing disorders or patients that are unassessable; denominator: all patients with IS, ICH, or UND);* thrombolytic therapy* (numerator: number of stroke patients treated with rt-PA; denominator: all patients with IS aged 18–80 years);* treatment on a SU* (numerator: number of stroke patients treated on a SU; denominator all patients with IS, ICH, or UND);* antiplatelet therapy* (numerator: number of patients receiving antiplatelet therapy during hospital stay or at discharge; denominator: all patients with IS alive at discharge and without anticoagulant treatment); and* anticoagulant therapy* (numerator: number of patients treated with anticoagulants at discharge or recommended at discharge [Poland without recommendation at discharge]; denominator: all patients with IS and AF alive at discharge).

### 2.4. Statistical Analysis

For estimating the probability of adhering to a specific quality indicator over time within a respective audit, multivariable logistic regression analyses were performed. Analyses were adjusted for age (age group), sex, stroke subtype (if applicable), day of admission (weekend versus weekday), AF, and level of consciousness on admission (ADSR, POLKARD, Riks-Stroke) or within the first week (NSSA). For the adjusted point estimates, corresponding 95% confidence interval was calculated. The reference category was the year in which information for the respective quality indicator was available for the first time. To address a potential selection bias by nonmandatory participation in some of the audits, main analyses are based on centers participating continuously over the whole study period. As sensitivity analyses, performed calculations were repeated with inclusion of all centers during the assessed time period. The category “unassessable” for screening for dysphagia was not documented in all audits. Therefore, as sensitivity analysis for audits not documenting the category “unassessable” all analyses were repeated restricting patients with no disturbed level of consciousness.

For taking into account clustering of patients in centers with possibly different time trends in quality of care, mixed effects modelling with random effects was applied. Therefore a hierarchical logistic regression model with a random intercept for centers was calculated with the SAS procedure PROC GLIMMIX. The variance component of the random intercept represents the between-centers variance. For calculating the proportion of the total variance explained by the level 2 variable the concept of the variance partition coefficient (VPC) was used. The latent variables method introduced by Snijders and Bosker [18] was used to calculate the VPC. Statistical analyses were performed using the SAS 9.2 Software Package.

### 2.5. Standard Protocol Approvals, Registrations, and Patient Consents

The study was approved by the ethics committee of the Charité-Universitätsmedizin Berlin (EA4/097/10) and is registered by ethics committee of the Medical Faculty of the University of Würzburg (215/11).

## 3. Results

Between 2004 and 2009, 930,978 patients (range between audits: 31,723 to 660,070) from 1,170 centers (trusts, hospitals, health boards, or departments; range between audits: 18 to 712) were registered within the participating audits. The presented results were restricted on centers participating continuously over the whole study period. The percentage of centers with continuous documentation ranged from 22% to 100% between audits. Centers not participating continuously had smaller numbers of patients (data not shown). Overall, 542,112 patients (range between audits: 31,155 to 303,023) were documented within the 538 centers (range between audits: 18 to 208) participating continuously over the whole study period (Table 1). The baseline characteristic of these patients is shown in Table 2.

### 3.1. Variations in Quality of Care over Time

The main results of univariate and multivariable analyses are presented in Table 3. A constant increase in the probability of screening for swallowing disorders was observed within the ADSR, Riks-Stroke, and NSSA with substantially lower absolute numbers within the ADSR. There was no clear time trend in the SSCA from 2005 to 2008 for screening for swallowing disorders until an increase in 2009. Restricting analyses to patients without disturbances of consciousness did not change results substantially (data not shown). Rates of thrombolysis increased in ischemic stroke patients aged 18–80 years in Riks-Stroke and the ADSR over time with a higher absolute proportion of patients receiving thrombolytic therapy within the ADSR. No clear time trend in the rate of thrombolysis was seen for POLKARD. Probability of receiving Stroke Unit treatment increased continuously in SSCA, Riks-Stroke, and NSSA with highest relative increase in the NSSA. No clear temporal patterns in antiplatelet therapy were observed in most audits except for the ADSR where rates of antiplatelet therapy were constantly increasing since 2007. Anticoagulant therapy in patients with atrial fibrillation increased in the ADSR and Riks-Stroke, both with an increase starting from 2007 onwards. No clear temporal changes in proportions of patients receiving anticoagulation were observed for POLKARD, the SSCA, and NSSA. No substantial differences were observed in the participating audits regarding variations in the investigated quality indicators over time when all centers were included in the analyses (data not shown).

To account for between-centers variations, variance partition coefficients (VPCs) are reported in Table 3. Between-centers variations differ between the audits as well as between quality indicators. For example, between-centers variation is very low for antiplatelet and anticoagulant therapy but substantially higher for treatment on a SU for some audits.

## 4. Discussion

For the first time, individual data from five prospective national stroke audits in Europe were pooled and analyzed over a 3- to 6-year time period between 2004 and 2009 when the EIS project was over. In most of the audits, a general trend towards a better quality of stroke care defined by standardized quality indicators was documented over the study period, mainly for swallowing testing, thrombolytic therapy, and Stroke Unit treatment. However, there were also some performance measures, such as antiplatelet therapy or anticoagulation in IS patients with AF, where no substantial variations in quality of care over time were observed in most of the audits. In some audits, a steeper increase from a specific year onwards in the defined performance measures was detected that might exceed a general constant trend toward a better provision of quality of stroke care.

Only a few studies investigated up to now time trends in delivery of quality of care within established audits. Similar to our findings for Riks-Stroke and the ADSR, Ferrari et al. found a consistent increase of thrombolysis rates from 4.9% in 2003 to 18.3% in 2011 within the Austrian stroke registry [7]. The Registry of the Canadian Stroke Network (RCSN) reported slightly higher thrombolysis rates compared to our findings (14.0%, 2003–2005 [15], and 15.7%, 2003–2008) [19]. Saposnik et al. reported a similar prescription rate of antiplatelets at discharge (92.7%) within the RCSN compared to our study [15]. The only difference to our analysis is that the RCSN included oral anticoagulants in their definition for antiplatelets. Similar anticoagulation rates were found within the Danish Stroke Registry (2003–2011) where 41.5% had been prescribed anticoagulants and 35.7% were registered as having a contraindication [16]. The proportion of patients being tested for swallowing disorders also substantially varied in previous studies. Some studies such as the PCNASR and the RCSN found lower screening rates for swallowing disorders (45.5%, 2001–2004; 56.7%, 2005–2007; and 56.0%, 2003–2005, resp.) compared to our data, except the ADSR [12, 15]. The PCNASR excluded patients who were on “nothing per oral.” Higher rates of assessment of swallowing disorders than in our study were reported from the population-based South London Stroke Register (SLSR), where 90.4% of the patients got a screening for swallowing disorders in 2004–2006 and 87.9% between 2007 and 2009 [20]. The SLSR observed also slightly higher SU admission rates between 2004 and 2006 of 76.4% and between 2007 and 2009 of 78.4% compared to the data from the NSSA [20].

There might be a number of potential reasons causing temporal changes of quality of acute stroke care within the different countries such as introduction of a new health care policy or new guidelines. For example, testing for swallowing disorders was implemented as quality indicator within the ADSR the first time in 2006 [21] and its documentation within Riks-Stroke in Sweden started in 2007 [22]. A similar increase of performance could be observed in both stroke audits after start of the documentation, but the ADSR started on a substantially lower level. This increase might reflect a better performance of the individual hospitals due to drawing attention to this measure. However, the increase might also be caused by a better documentation of hospitals within the audits, which might be perceived as improvement of quality of care in itself. However, also other methods of implementation, for example, better finance for stroke services and restructuring of stroke services, might have been reasons for improvement of quality of care [11]. The UK National Stroke Strategy 2007 recommended screening for dysphagia within 24 hours [23]. A consistent increase for screening for dysphagia has been reported within the NSSA already since 2006. The largest increase for thrombolysis was documented in the Swedish audit from 2006 onwards. Accordant to this finding, the Swedish acute care guidelines from 2005 recommended thrombolysis as an important part of the treatment of stroke patients [24]. The National Stroke Strategy in the UK recommended in 2007 the timely access for 75% of stroke patients to an acute Stroke Unit [23]. This figure was already nearly achieved in 2008 based on the audit data (72.8%). Consistently high levels of antiplatelet therapy were observed within the NSSA. The proportion of about 97% of patients treated with antiplatelets at discharge in the NSSA might represent a ceiling effect with the highest possible treatment proportion. The 2004 National Clinical Guideline for Stroke already recommended the prescription of antiplatelet agents for all patients with ischemic stroke or TIA who are not on anticoagulation [25]. The substantial increase in uptake of antiplatelet therapy in ischemic stroke patients in the ADSR between 2005 and 2007 may be explained by the revised guideline on the topic of diagnostic and therapy in neurology in 2005 that recommended also antiplatelets in the early stage after stroke [26]. The between-centers variation was mostly low, except for Stroke Unit treatment. Differences might be partly explained by voluntary participation in some of the audits (ADSR, POLKARD).

The study has limitations. Within the study, data from existing audits across Europe were analyzed. In contrast to other initiatives [27], there was no agreement on a common set of variables or a common method of data collection before the study took place. In addition, data were collected using different approaches (e.g., documentation of consecutively admitted patients by staff members versus chart review for defined sample of patients). Therefore, we cannot exclude that variations in data collection methods might have introduced some information bias in our analyses. There was no complete or no comparable information over time for some of the investigated quality indicators across all audits. In some of the audits (e.g., ADSR or POLKARD), there were no formal checks established for estimating completeness of case ascertainment neither within a center nor of centers included within a country as the participation of hospitals within the audit initiatives was voluntary. Therefore, we cannot exclude that some of the findings were caused by selection biases rather than real changes in quality of care provided, for example, by variations in case mix or patient selection across participating centers and countries. To maintain data accuracy, all of the audits used data validation tools such as plausibility checks. Additionally, source data verification was conducted by some of the audits. No outcome data such as death or disability at 90 days could be calculated due to the limited availability within the audits. It remains unclear if the increasing number of patients per center observed in some of the audits might be caused by a more complete documentation within the center or by increasing admission rates within the participating centers. However, restricting analysis to centers with complete information over the whole time period yielded similar estimates compared to including all centers in the analyses. We had only very limited information regarding the characteristics of the participating centers within the audits. Therefore, we were not able to investigate the potential influence of the center characteristics on time trends of appropriate care delivery. Unfortunately, we did not have comparable information on stroke severity based on standardized instrumental scales across audits because the audits used different scales for assessing stroke severity (e.g., NIHSS, Glasgow coma scale, and level of consciousness). We therefore used level of consciousness as proxy for adjusting for stroke severity. Furthermore, data were derived between 2004 and 2009 and the situation may have changed since then.

## 5. Conclusions

Heterogeneous patterns were identified in the five participating European audits regarding trends in quality of care over time. A general trend towards a better quality of stroke care provided over the study period was observed in most audits. The introduction of a specific performance measure within an audit activity might contribute to improved quality of acute stroke care provided over time.

## Figures and Tables

**Table 1:** Characteristics of participating audits, 2004–2009^*∗*^.

	ADSR	POLKARD	SSCA	Riks-Stroke	NSSA
Number of centers^#^	193	45	18	74	208
No. of patients per center per year, median (IQR)					
2004	143 (75–372)	205 (160–264)	0	256 (169–435)	40 (37–40)
2005	164 (80–360)	141 (76–188)	369 (225–514)	277 (183–426)	0
2006	0	0	269 (187–501)	270 (170–418)	66 (48–79)
2007	217 (85–456)	280 (168–342)^†^	339 (188–514)	261 (165–423)	0
2008	253 (79–535)	51 (33–69)^†^	382 (210–578)	269 (165–399)	59 (50–60)
2009	346 (86–616)	0	297 (191–601)	268 (177–427)	0

^*∗*^Center participating continuously over the whole study period only; patients with missing center allocation or with missing/default year of admission were not considered; ^#^number of centers referring to participating center, trusts, or departments; IQR: interquartile range; ^†^no complete calendar years of documentation.

**Table 2:** Patient and clinical characteristics, 2004–2009, centers participating continuously over the whole study period^*∗*^.

	ADSR	POLKARD	SSCA	Riks-Stroke	NSSA
	(*n* = 303,023)	(*n* = 31,564)	(*n* = 34,962)	(*n* = 141,389)	(*n* = 31,174)
Age, median (IQR)	75 (66–82)	73 (62–80)	75 (66–83)	78 (69–84)	78 (69–85)
Female sex, %	49.4	50.9	51.3	49.3	52.5
Dependency prestroke, %					
Independent	77.4	85.0	84.9	85.0	77.4
Dependent	22.5	15.0	15.1	15.0	22.6
Stroke subtype, %					
Ischemic stroke	85.1	86.6	82.7	84.1	74.3
Intracerebral haemorrhage	9.0	10.5	10.6	11.8	11.7
Unknown/undefined	5.9	2.9	6.7	4.1	14.0
Interval onset-admission, %					
<3 hours	29.3	19.3	#	25.2	19.7
>3 hours/missing	70.7	80.7	#	74.8	80.3
Day of admission, %					
Weekday	75.7	75.8	75.1	74.9	74.1
Weekend	24.3	24.2	24.9	25.1	25.9
Atrial fibrillation, %					
Yes	25.0	22.4	22.7	28.3	20.0
No	75.0	77.6	77.3	71.7	80.0
Level of consciousness, %^†^					
Awake	83.7	72.9	#	81.5	62.9
Disturbed (drowsy to coma)	16.3	27.1	#	18.5	37.1

^*∗*^Analyses were restricted to patients with IS, ICH, or UND and without missing values in the respective variables; IQR: interquartile range; ^#^variable not documented or not documented in a comparable way; ^†^level of consciousness on admission or during first day except the NSSA, here worst level of consciousness during first week.

**Table 3:** Variations and changes in quality of care over time, 2004–2009, center participating continuously over the whole study period^*∗*^.

	2004		2005		2006		2007		2008		2009		VPC^‡‡^
	%	OR (95% CI)	%	OR (95% CI)	%	OR (95% CI)	%	OR (95% CI)	%	OR (95% CI)	%	OR (95% CI)	
Swallowing test done^†^													
ADSR	#	#	#	#	#	#	52.9	1.00	63.7	1.65 (1.60–1.70)	70.3	2.44 (2.37–2.52)	0.3847
POLKARD	#	#	#	#	#	#	#	#	#	#	#	#	#
SSCA	#	#	74.9	1.00	78.4	1.10 (0.99–1.21)	73.3	1.02 (0.93–1.12)	72.4	0.88 (0.80–0.96)	83.8	1.94 (1.75–2.15)	0.2697
Riks-Stroke	#	#	#	#	#	#	84.1	1.00	89.1	1.65 (1.55–1.76)	92.7	2.68 (2.50–2.86)	0.0851
NSSA	75.4	1.00	#	#	77.2	1.08 (1.01–1.16)	#	#	81.2	1.45 (1.34–1.56)	#	#	0.1263
Thrombolysis^‡^													
ADSR	6.0	1.0	7.1	1.20 (1.11–1.30)	#	#	8.6	1.57 (1.45–1.69)	9.5	1.70 (1.58–1.82)	11.2	2.04 (1.90–2.19)	0.2794
POLKARD	0.9	1.0	0.6	0.70 (0.43–1.14)	#	#	1.5	1.85 (1.33–2.57)^††^	1.2	1.83 (1.05–3.17)^††^	#	#	0.3866
SSCA	#	#	#	#	#	#	#	#	#	#	#	#	#
Riks-Stroke	2.2	1.0	3.4	1.54 (1.31–1.82)	3.6	1.66 (1.41–1.96)	5.6	2.60 (2.24–3.03)	7.0	3.27 (2.82–3.79)	7.9	3.74 (3.24–4.32)	0.0824
NSSA	#	#	#	#	#	#	#	#	#	#	#	#	#
Treatment Stroke Unit^§^													
ADSR	#	#	#	#	#	#	#	#	#	#	#	#	#
POLKARD	86.7	1.0	91.3	1.99 (1.75–2.27)	#	#	90.0	1.71 (1.54–1.89)^††^	91.2	1.90 (1.59–2.27)^††^	#	#	0.5592
SSCA	#	#	66.2	1.0	72.7	1.25 (1.15–1.36)	76.0	1.49 (1.36–1.62)	74.6	1.36 (1.25–1.48)	78.1	1.71 (1.57–1.87)	0.3382
Riks-Stroke	77.4	1.0	78.0	1.07 (1.02–1.12)	81.1	1.30 (1.24–1.37)	82.1	1.38 (1.31–1.45)	84.0	1.58 (1.50–1.66)	86.8	2.04 (1.94–2.15)	0.2299
NSSA	45.6	1.0	#	#	61.4	2.46 (2.30–2.64)	#	#	72.8	4.07 (3.78–4.37)	#	#	0.3745
Antiplatelet therapy^||^													
ADSR	91.4	1.0	91.4	0.98 (0.92–1.05)	#	#	95.5	1.92 (1.77–2.08)	96.4	2.35 (2.17–2.55)	97.0	2.73 (2.52–2.96)	0.0964
POLKARD	93.4	1.0	93.7	1.10 (0.93–1.30)	#	#	94.1	1.18 (1.03–1.36)^††^	88.8	0.68 (0.56–0.82)^††^	#	#	0.1520
SSCA	#	#	85.3	1.0	87.8	1.24 (1.08–1.42)	88.8	1.38 (1.21–1.58)	90.7	1.72 (1.49–1.98)	88.1	1.30 (1.14–1.48)	0.0239
Riks-Stroke	91.7	1.0	91.3	0.95 (0.87–1.03)	91.4	0.96 (0.88–1.04)	92.1	1.06 (0.97–1.15)	91.6	0.99 (0.91–1.08)	91.5	0.97 (0.90–1.06)	0.0239
NSSA	97.7	1.0	#	#	96.4	0.67 (0.51– 0.86)	#	#	97.6	1.04 (0.80– 1.36)	#	#	0.21790
Anticoagulant therapy^**∗∗**^													
ADSR	37.7	1.0	36.6	0.99 (0.92–1.07)	#	#	49.6	1.82 (1.69–1.96)	52.2	2.06 (1.91–2.21)	55.3	2.30 (2.15–2.47)	0.1014
POLKARD	26.0	1.0	26.4	0.99 (0.82–1.21)	#	#	25.0	0.94 (0.80–1.11)^††^	20.5	0.76 (0.58–1.01)^††^	#	#	0.1436
SSCA	#	#	39.5	1.0	38.6	0.89 (0.69–1.15)	41.0	1.10 (0.83–1.44)	37.8	0.93 (0.70–1.24)	30.7	0.88 (0.66–1.18)	0.1297
Riks-Stroke	32.5	1.0	31.6	0.96 (0.87–1.05)	31.0	0.95 (0.87–1.05)	33.0	1.09 (0.99–1.20)	36.1	1.24 (1.13–1.36)	37.2	1.37 (1.25–1.51)	0.0556
NSSA	37.6	1.0	#	#	32.9	0.86 (0.70–1.05)	#	#	34.3	0.94 (0.77–1.15)	#	#	0.0456

^*∗*^Analyses were restricted to patients without missing values; % refers to patients adhering to the specific quality indicator; OR: odds ratio; CI: confidence interval; OR and 95% CI were derived from a logistic regression model adjusted for age, sex, stroke subtype (if applicable), day of admission, AF, and level of consciousness; ^#^not documented or not documented in a comparable way or no documentation in the respective year; ^†^all patients with IS, ICH, or UND and patients with swallowing test done and unassessable status; ^‡^In IS only, age between 18 and 80 years; ^§^all patients with IS, ICH, or UND; ^||^anytime during stay or at discharge in patients with IS alive at discharge and without anticoagulant therapy; ^*∗∗*^anytime during stay or at discharge or recommended at discharge in patients with IS and AF alive at discharge; ^††^POLKARD no complete calendar year of documentation; ^‡‡^variance partition coefficient, to account for between-centers variation within the audits.
